# Supplementary material for: Upcycling rice yield trial data using a weather-driven crop growth model
Source: Commun Biol. 2023 Jul 21;6:764. doi: 10.1038/s42003-023-05145-x (PMC10362053; doi:10.1038/s42003-023-05145-x)
Supplement: Supplementary file 2 — Supplementary Figures [file 42003_2023_5145_MOESM2_ESM.pdf]

Fig.S1. Shimono

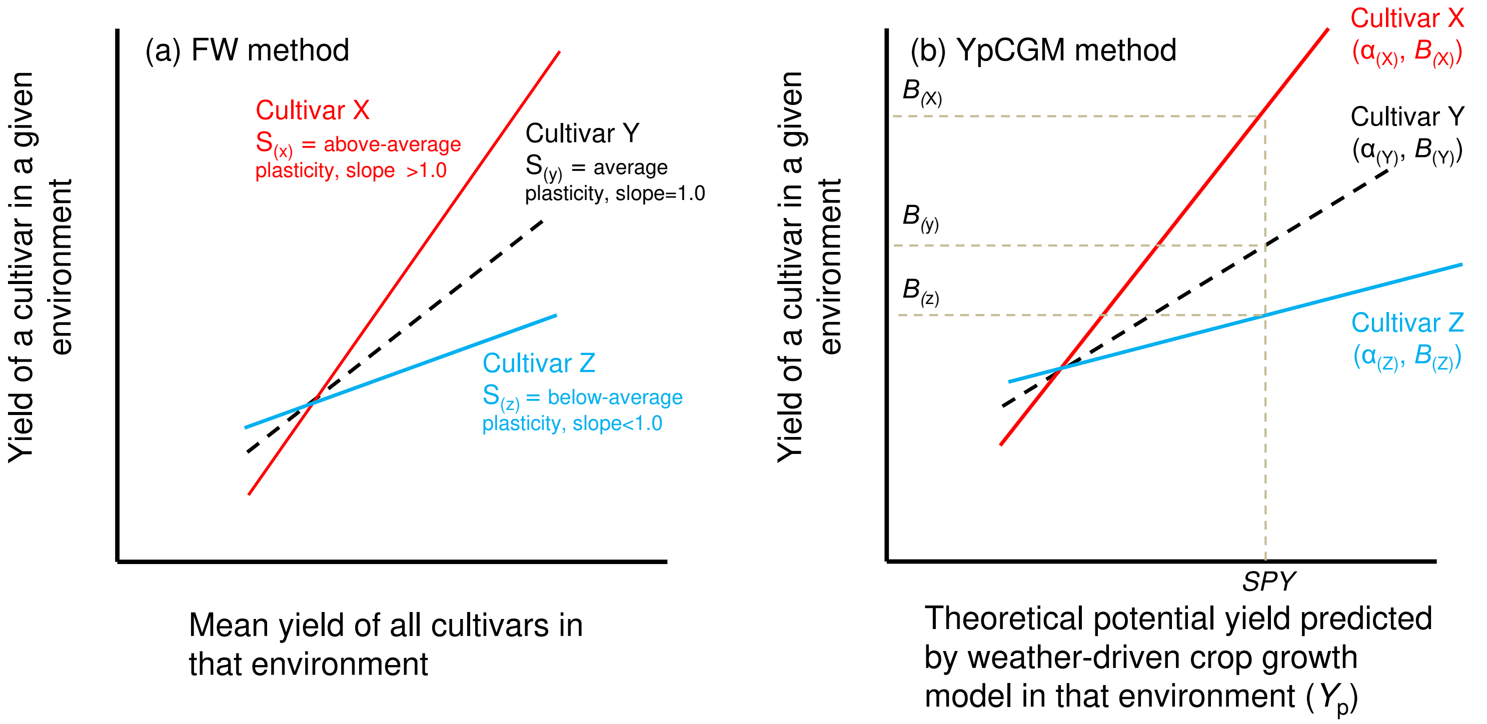

$$Y_{obs(i)} = \alpha_{(i)}(Y_{p(i,\,j)} - SPY) + B_{(i)} \quad (eq1.)$$

Supplementary Figure S1. (a) Illustration of the Finlay and Wilkinson (1963) regression method (the FW method) and (b) the new method developed here using the potential yield (Y_p) predicted by a weather-driven crop growth model (the YpCGM method) to account for the genotype × environment interaction and its effects on yield. The FW method is limited to yield data measured under the same experimental conditions in a side-by-side comparison. The YpCGM method combines data from many experiments under different conditions using the Y_p calculated from weather data by using the yield-plasticity (α; dimensionless) and the yield-ability (β; t/ha), *SPY* is a standardized potential yield = 8 t/ha (eq. 1). $Y_{obs(i,\,j)}$ and $Y_{p(i,\,j)}$ represent the observed and potential yield of a given cultivar (i) in a given environments (j) (year, location, and management regime).

Fig.S2. Shimono

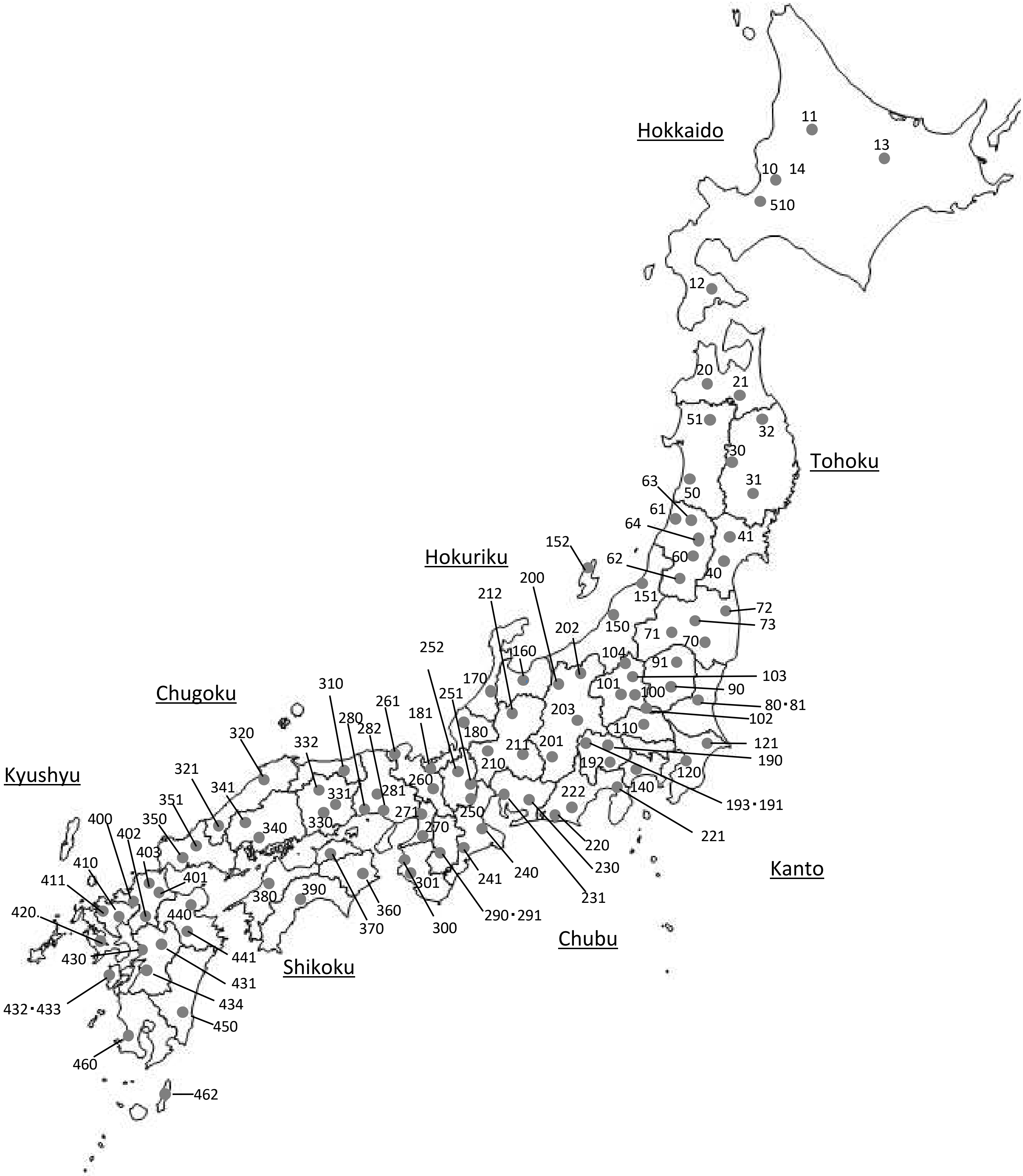

Supplementary Figure S2. The 110 locations in Japan from which yield trial data were sourced. Numbers refer to the locations in Supplementary Table S1. Map was illustrated by Haku-Chizu II (Mapquest, Aichi, Japan).

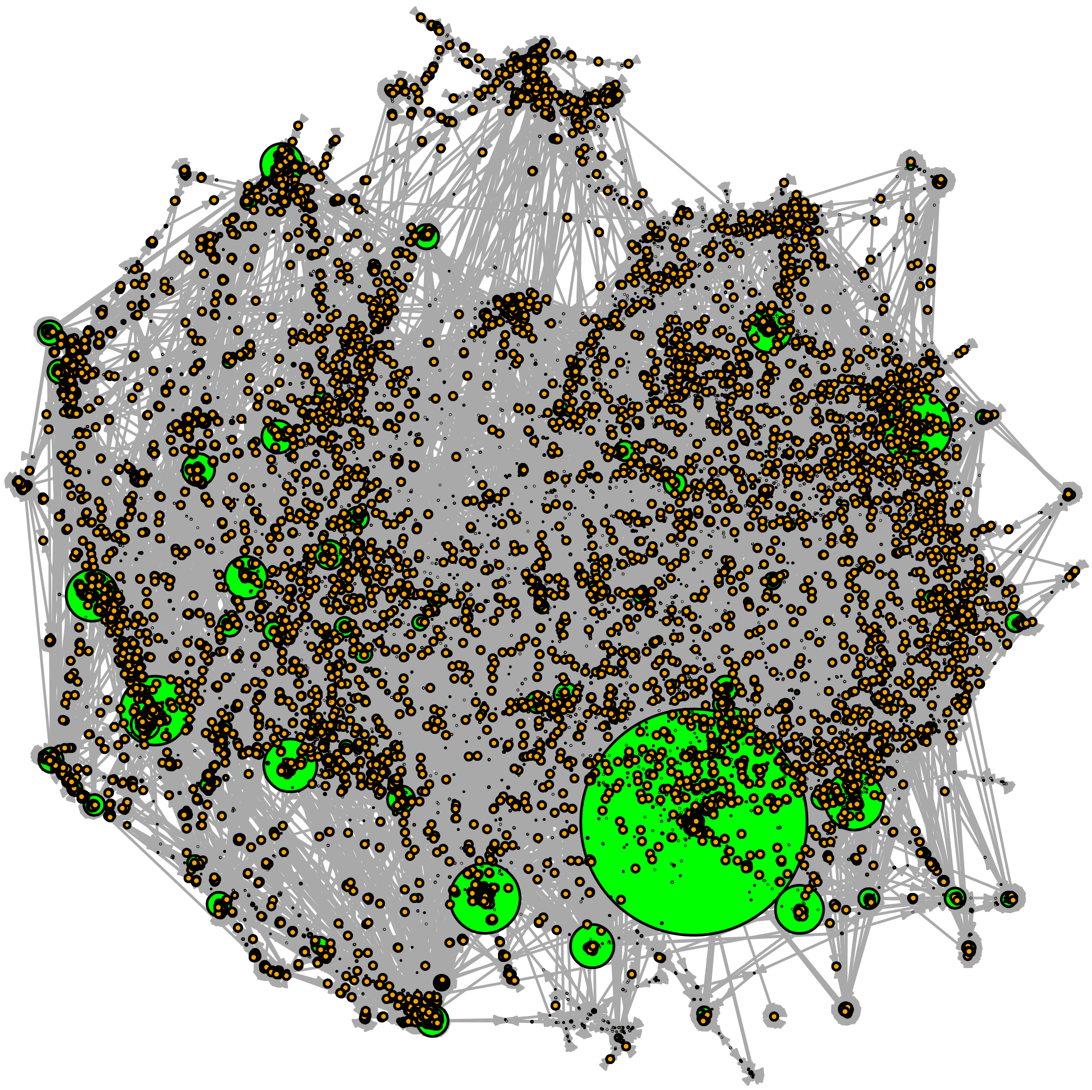

Supplementary Figure S3. Pedigree matrix classification using the k-medoids method (k = 200 clusters) for the 14 032 rice cultivars for which pedigree information was available. We classified the data into groups with k = 200 and selected the central node as representative cultivars. The cultivars selected in this way are represented by green area. The arrows coming from each node connect from a parent accession to a child accession (i.e., they represent the number of progeny), and the size of the circle that represents a node is proportional to the number of progeny. The largest circles were selected as representative accessions and are relatively evenly distributed within the network.

Fig.S4. Shimono

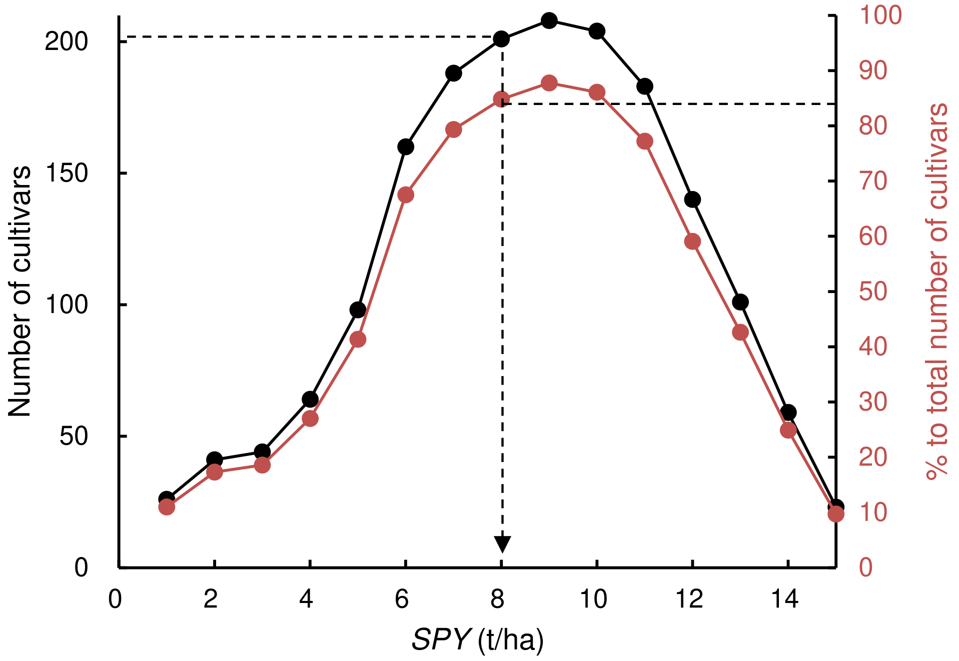

Supplementary Figure S4. Number of cultivars covered for the standardized potential yield (*SPY*) within the range of experienced Y_p, and the percentage to total 237 cultivars. Here, we set 8t/ha as *SPY*.

Fig.S5. Shimono

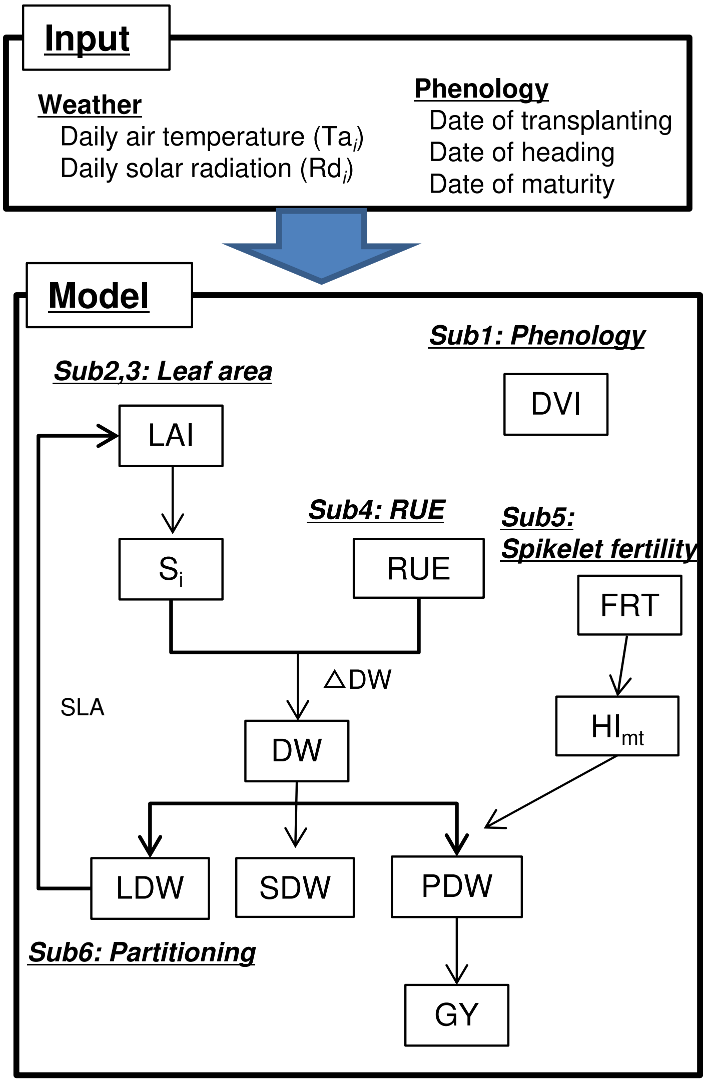

Main (Horie, 1987)

$$DW_i = DW_{i-1} + \triangle DW$$
$$\triangle DW_i = RUE_i \times S_i$$
$$S_i = 1 - EXP(-0.4 \times LAI_i)$$

Sub1: Phenology(New)

From transplanting to heading (0.5<DVI ≤ 2.0)

$$DVIi = 0.5 + \sum_{t=1}^{i} DVR_{i-1}$$
$$DVR_i = (2.0 - 0.5) \times (Ta - 10) / SumT_{tp\text{-}hd}$$
$$SumT_{tp\text{-}hd} = \sum_{date-tp}^{date-hd} dT$$

dT = Ta – 10, where Ta>10

dT= 0, where Ta≤ 10

From heading to maturity(2.0< DVI ≤ 3.0)

$$DVIi = DVI_{i-1} + \sum_{t=1}^{i} DVR_{i-1}$$
$$DVR_i = (Ta - 10) / SumT_{hd\text{-}mt}$$
$$SumT_{hd\text{-}mt} = \sum_{date-hd}^{date-mt} dT$$

dT = Ta – 10, where Ta>10

dT= 0, where Ta≤ 10

Sub2: Leaf area increase (Horie, 1987)

From transplanting to heading (DVI≤ 2.0)

$$LAI_i = [LAI_{i-1} \times EXP(\frac{1}{F}\frac{dF}{dt})]$$

$\frac{1}{F}\frac{dF}{dt}$= 0.247 [1.0 – EXP (-0.07(Ta –11.5))] [1.0- $(LAI_i/6.0)^{0.723}$],
where Ta> 11.5

$\frac{1}{F}\frac{dF}{dt}$ =0, where Ta≤ 11.5

Sub3: Leaf senescence (Shiomono et al., 2007)

From heading to maturity (2.0< DVI ≤ 3.0)

$$LAI_i = SLA_{hd} \times LDW_i$$
$$SLA_{hd} = LAI_{hd} / LDW_{hd}$$

Sub4: RUE (Shiomono et al., 2007)

RUEi = 2.0, where DVI<2.0

RUEi = 2.0×(1 +0.001)/[1 + 0.001 × EXP {((DVI_i ×2/3)-1)/0.1},
where 2.0< DVI ≤ 3.0

Sub5: Spikelet fertility (Shimono et al., 2007)

$$FRT = 100 - 94.1/[1 + EXP(3.78 - 0.0513 \times CDD)]$$
$$CDD = \sum_{DVI=1.0}^{DVI=2.0} CDi$$

CDi = (20 – Ta), where 22_e>Ta

CDi = 0, where 22≤ Ta

Sub6: Partitioning (Shimono et al., 2007)

$$LDW = VDW \times [(-0.161 \times DVI_i) + 0.6]$$

VDW = TDW – PDW, where 2.0≦ DVI ≤ 3.0

PDW = TDW × RPW, where 2.0≦ DVI ≤ 3.0

$$GY = (RPW - 0.120)/0.0102/100 \times TDW$$

$$RPW = 0.578 \times (DVI - 1.79)$$

($RPW < RPW_{max}$, if not $RPW = RPW_{max}$)

$$RPW_{max} = 0.0102\ HI_{mt} + 0.120$$

Abbreviation

DW= total dry weight (g m^{-2})
PDW= Panicle dry weight (g m^{-2})
VDW= Vegetative organ dry weight (g m^{-2})
LDW= Leaf dry weight (g m^{-2})
RPW=Ratio of panicle dry weight (g m^{-2})
RUE= Radiation use efficiency (g MJ^{-1})
FRT= Spikelet fertility (%)
HI_{mt} = Harvest index at maturity
LAI_i = Leaf area index at i-day
SLA_{hd} = Specific leaf area at heading (m^2 g^{-1})
LDW_{hd}= Leaf dry weight at heading (g m^{-2})
$T_{stop\text{-}minT}$ = Minimum temperature for growth during ripening stage (°C)=13

Supplementary Figure S5. Overview of crop growth model revised from Figure 1 in Masuya and Shimono (2017)

Grain yield

Fig.S6. Shimono (1/4 continue)

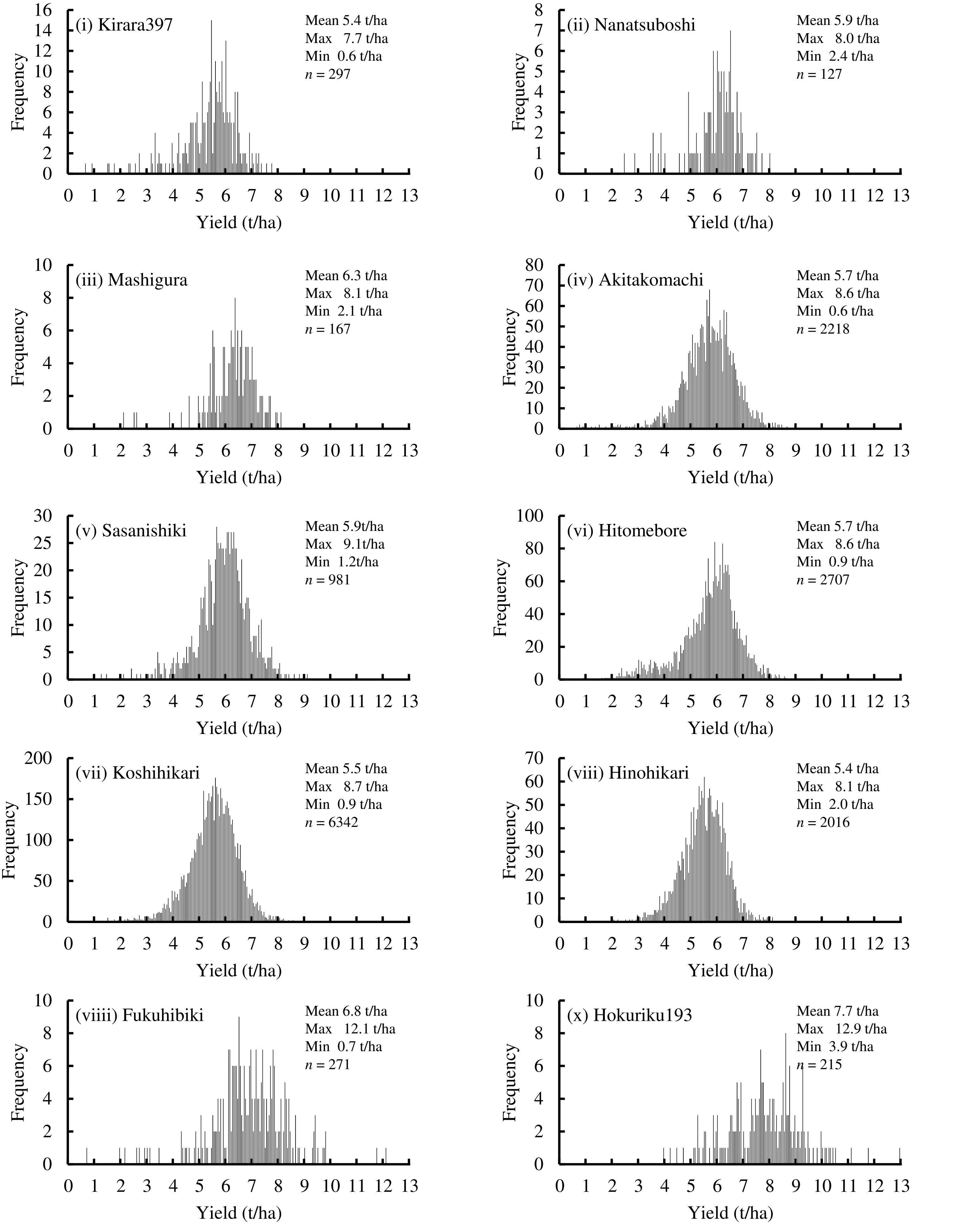

Days to heading

Fig.S6. Shimono (2/4 continue)

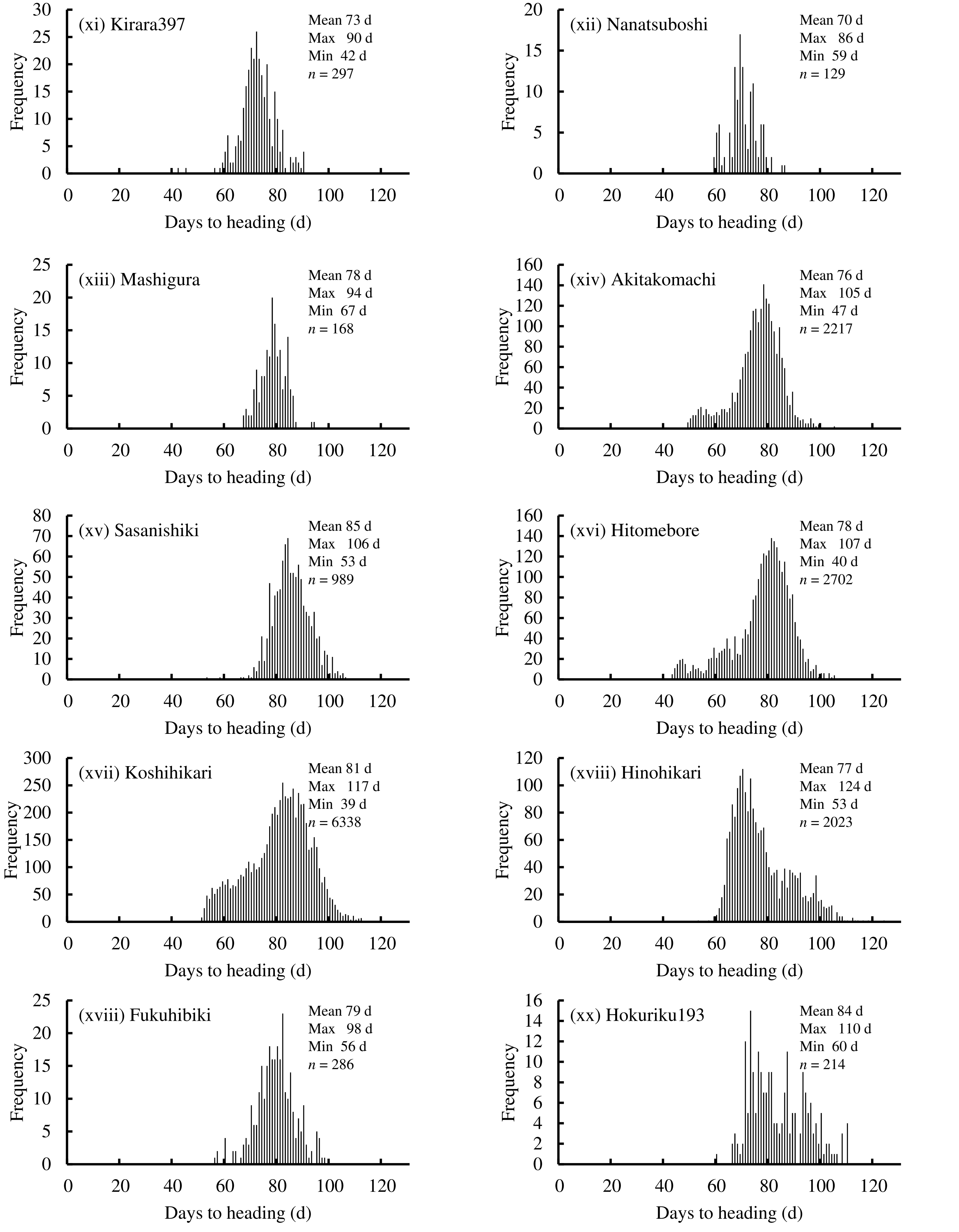

Panicle number

Fig.S6. Shimono (3/4 continue)

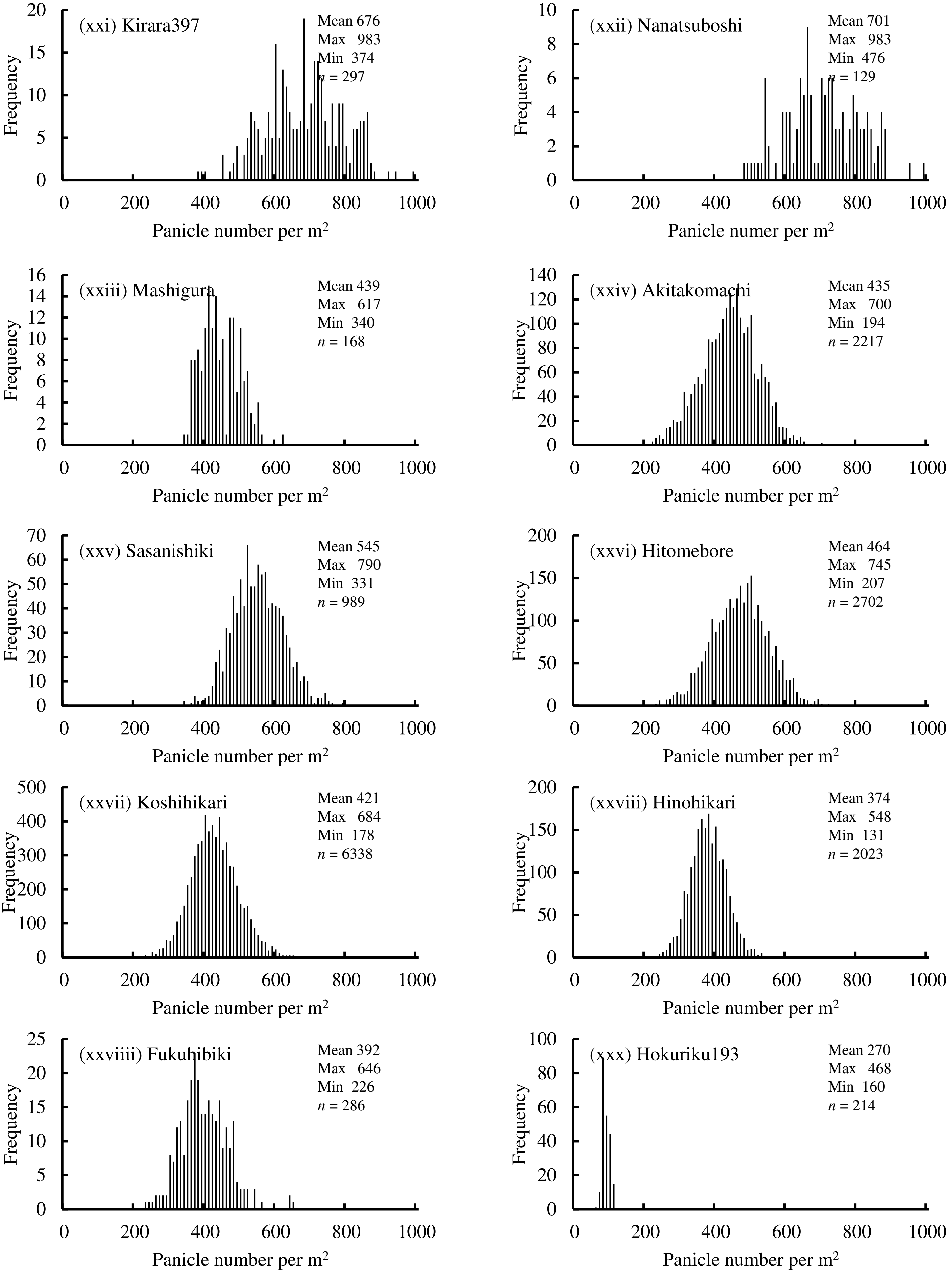

Panicle length

Fig.S6. Shimono (4/4 continue)

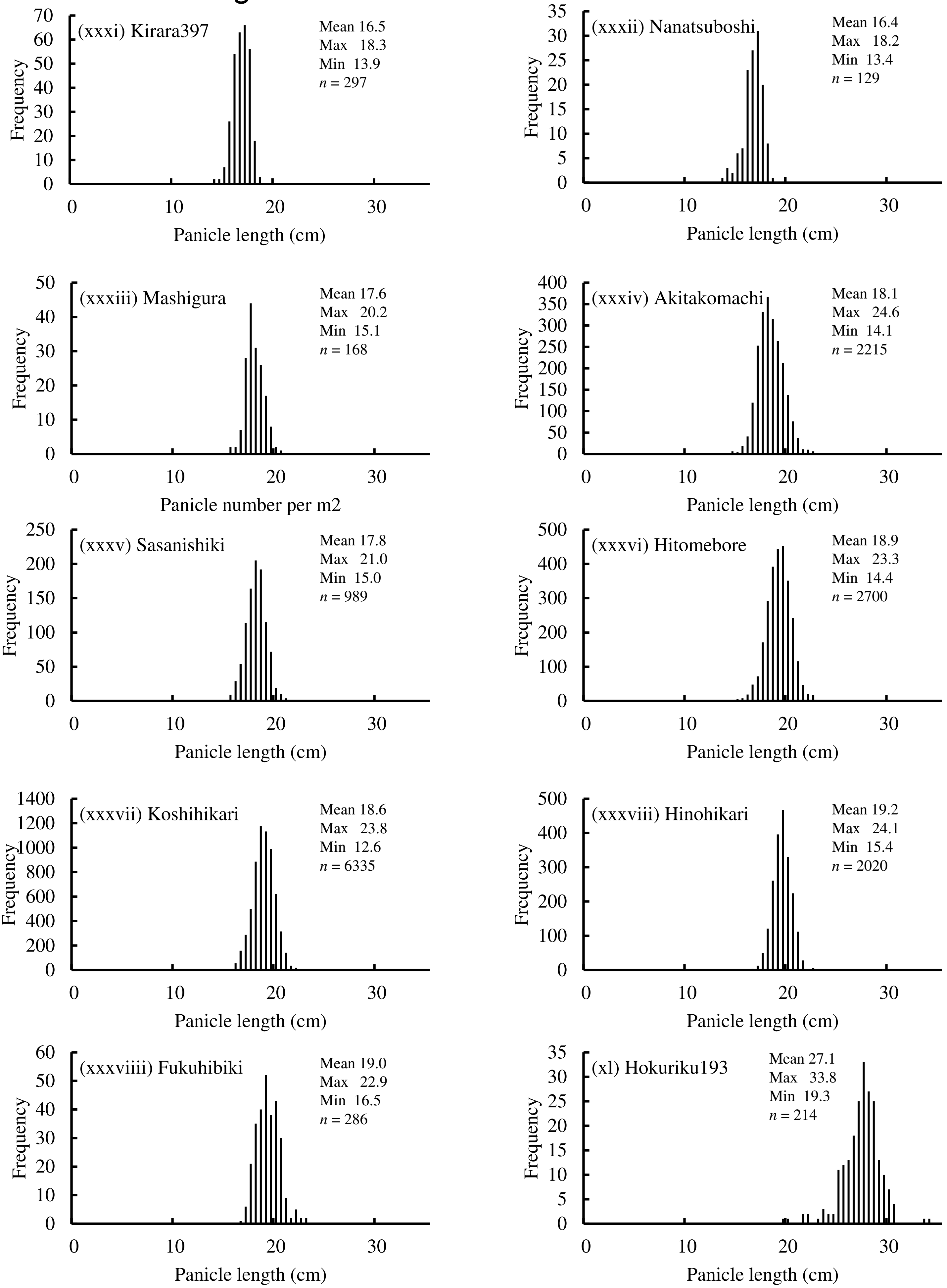

Supplementary Figure S6. Variation of the observed yield (i-x), days to heading (xi-xx), panicle number (xxi-xxx) and panicle length (xxxi-xl) of Japan's recent 10 major cultivars that together accounted for more than half of the area of cultivated rice from 2010 to 2020.

Obs. yield vs Y_p

Fig.S7. Shimono (1/3 continue)

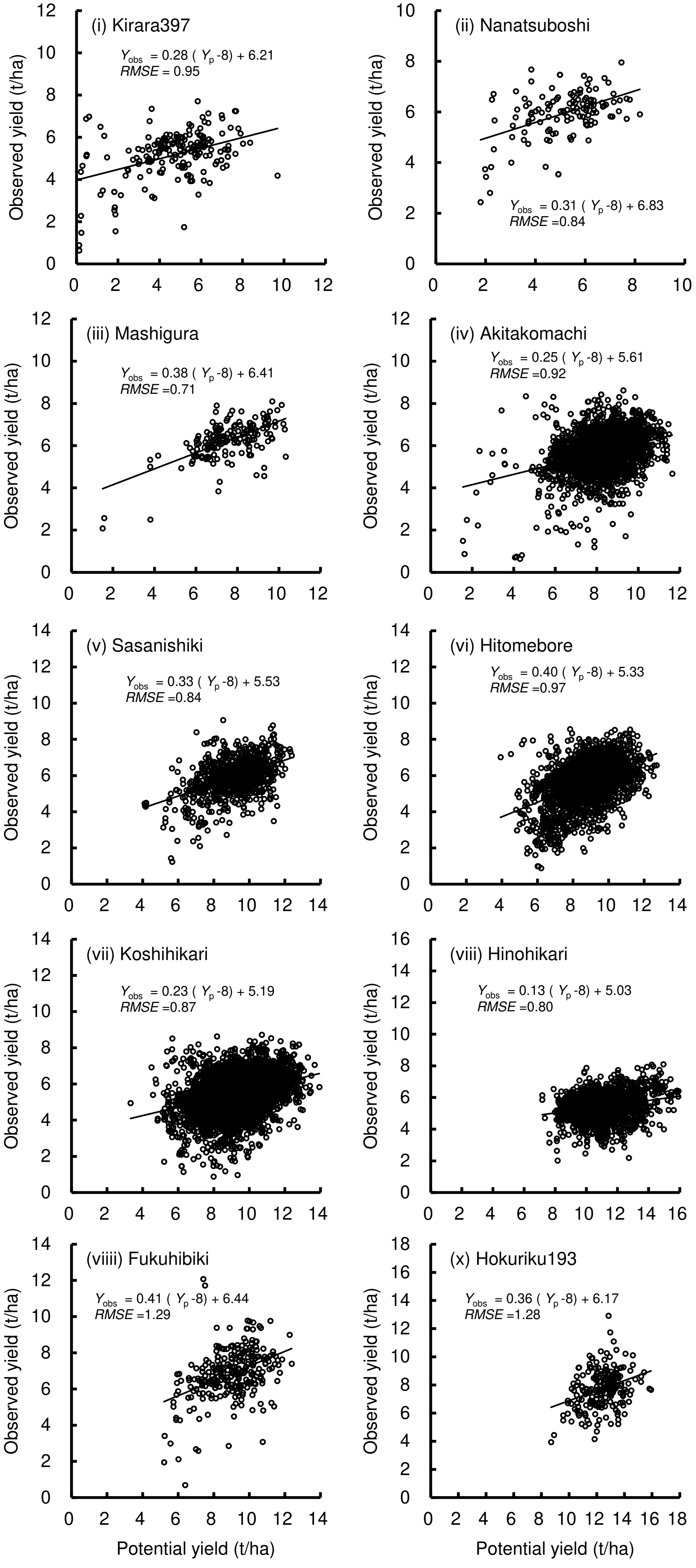

Obs. yield vs PN

Fig.S7. Shimono (2/3 continue)

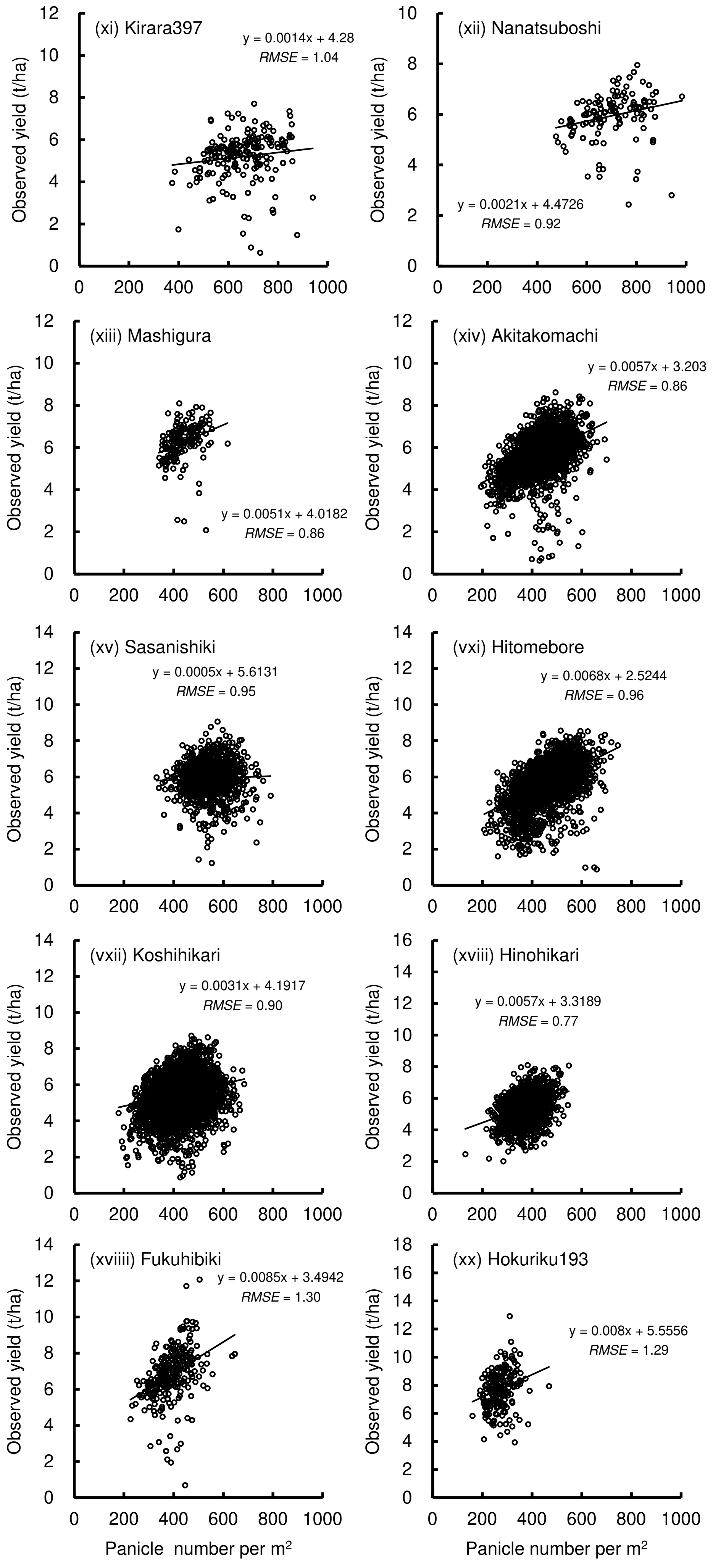

Obs. yield vs PL

Fig.S7. Shimono (3/3 continue)

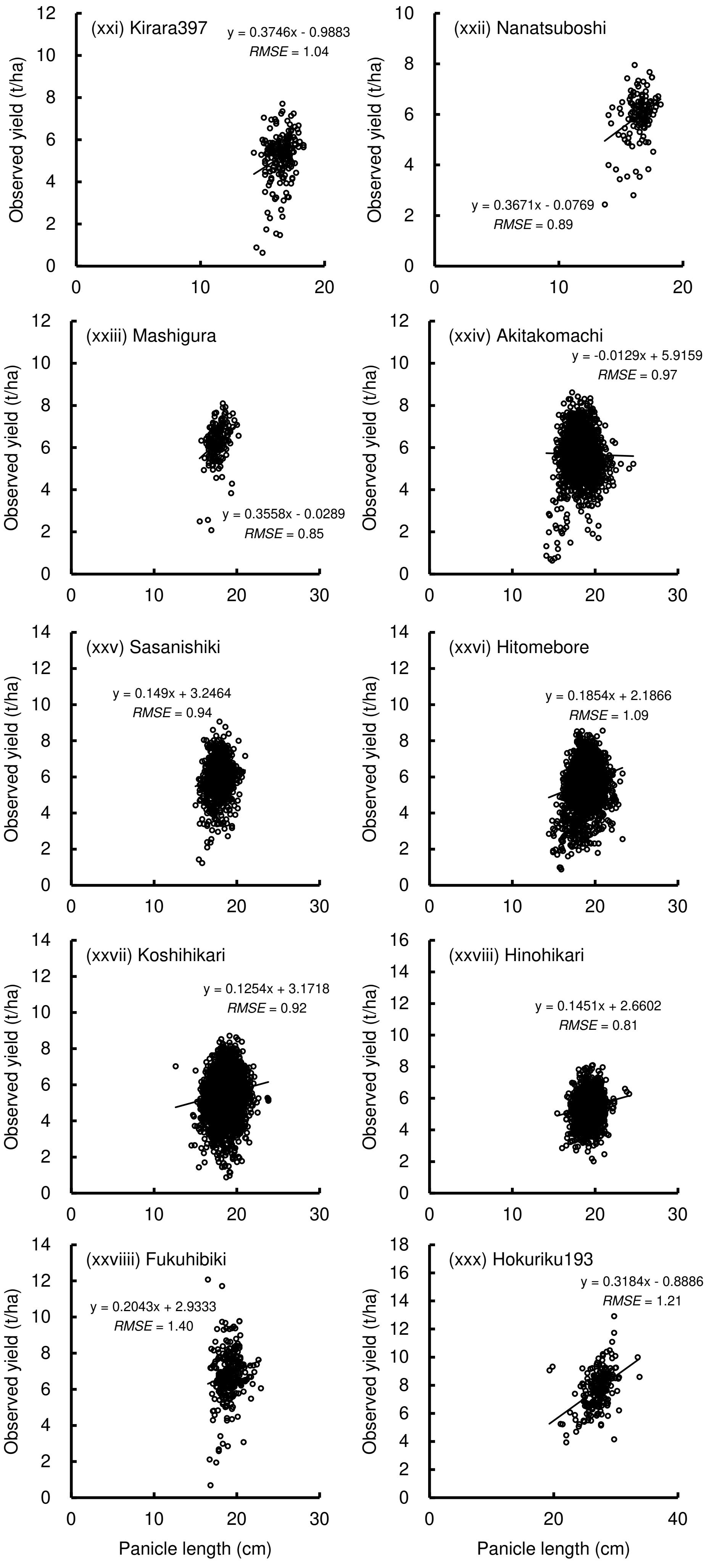

Supplementary Figure S7. Relationships of the observed yield against either potential yield (Yp) (i-x), observed panicle number (PN) (xi-xx) and observed panicle length (PL) (xxi-xxx) of Japan's 10 recent major cultivars estimated by using the weather-driven crop growth model. Root mean square error (*RMSE*).

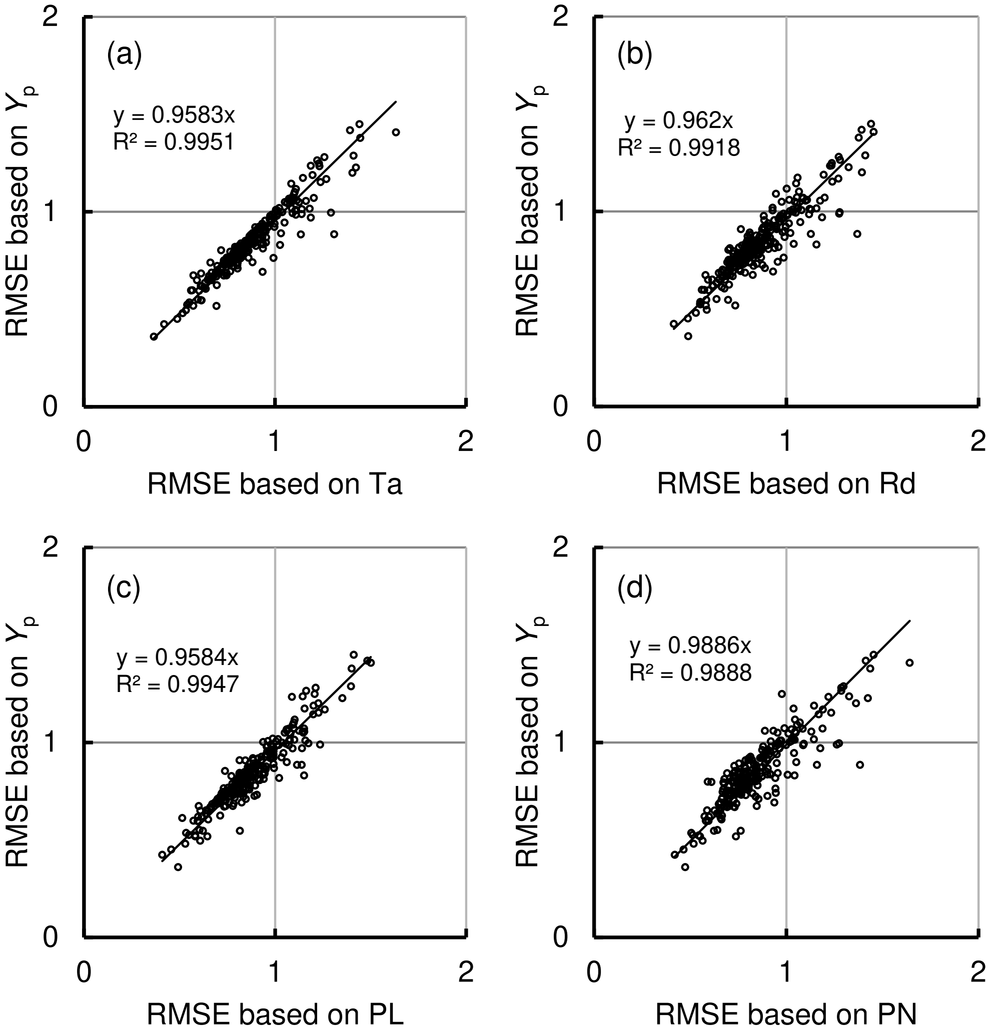

Supplementary Figure S8. Root-mean-square errors (*RMSE*) of the difference between the observed yield and the predicted yield based on the potential yield (Y_p) calculated by the weather-driven crop growth model, relative to the RMSE predicted by (a) accumulated air temperature (Ta), (b) accumulated solar radiation (Rd), (c) the observed panicle length (PL) and (d) the observed panicle number (PN) of the 237 core cultivars. *RMSE* of each cultivar was calculated independently from n = 20 to 6342 trials.

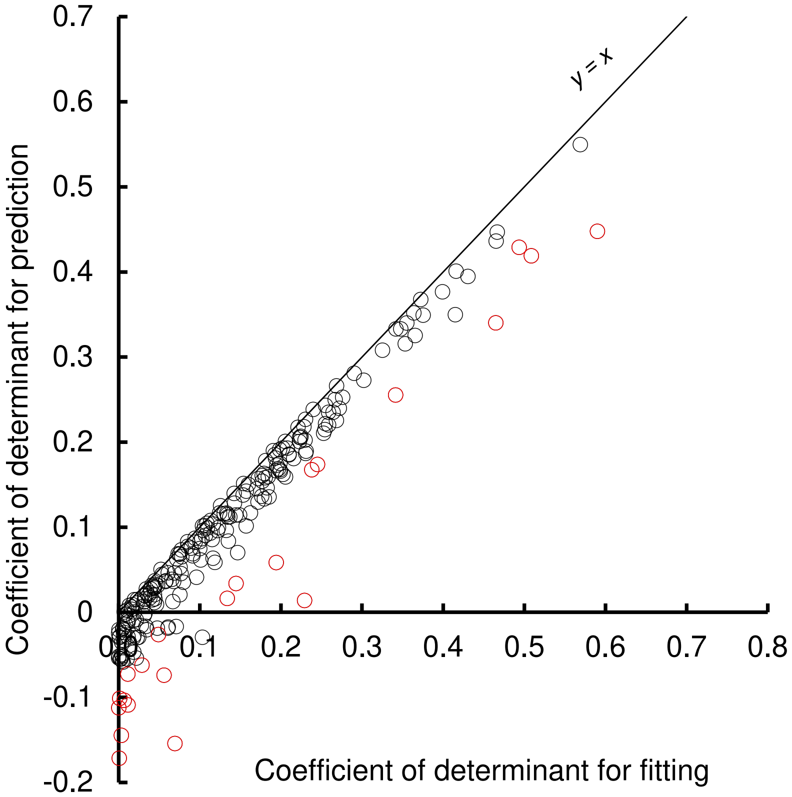

Supplementary Figure S9. Coefficients of determination of fitting (proportion of variation in fitted values to total variation) and coefficients of determination of prediction (proportion of variation in predicted values to total variation) for the 237 core rice cultivars. One point represents one cultivar. Predicted values of the cultivars were obtained via leave-one-out cross-validation. Points marked in red indicate cultivars for which the number of environments (number of data used in the regression model) is less than 50.

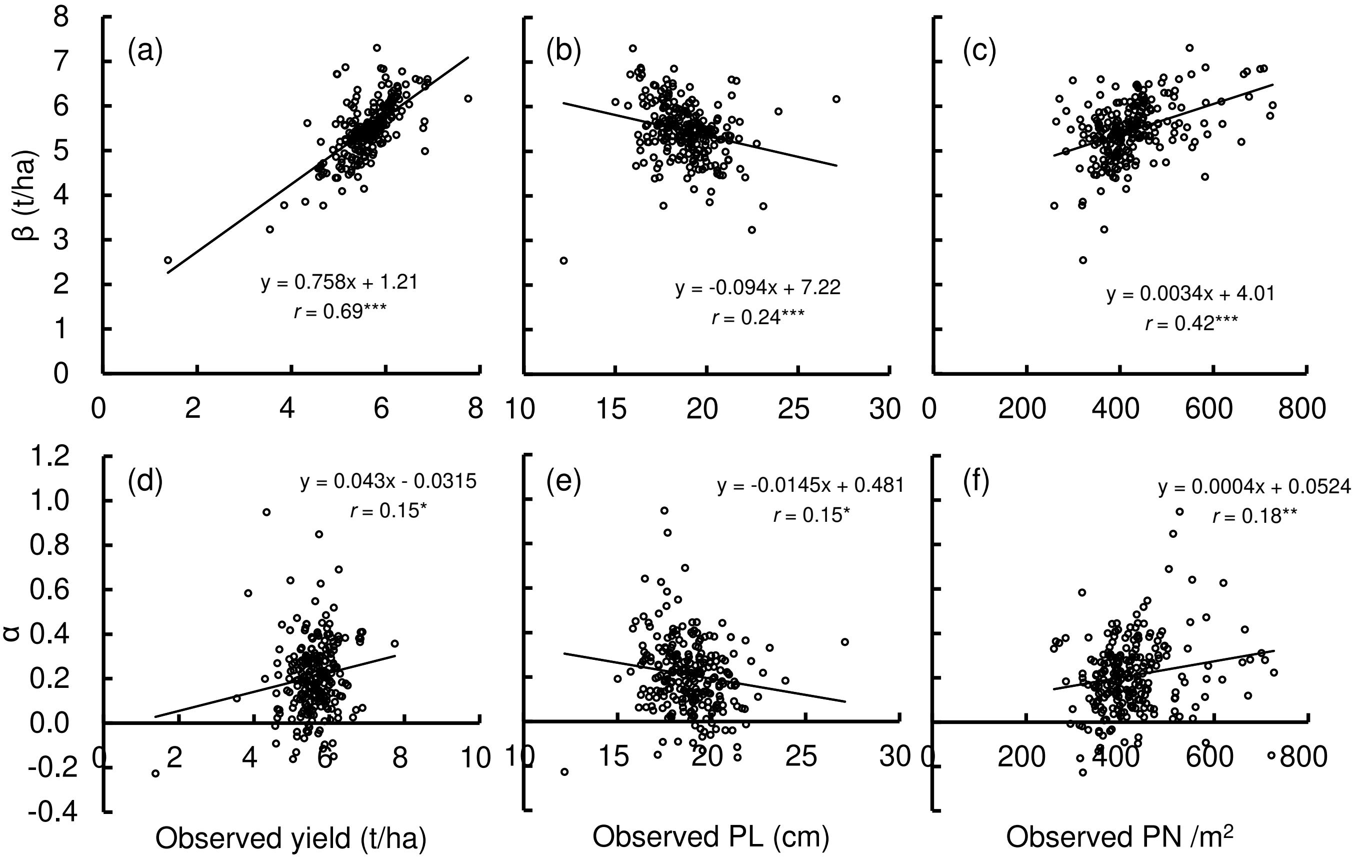

Supplementary Figure S10. Relationship of yield-ability (β) and its plasticity (α) against (a,d) the observed mean yield, (b,e) the observed mean panicle length (PL) and (c,f) the observed panicle number (PN) of the 237 core cultivars. *** P<0.001, ** P<0.01, * P<0.05

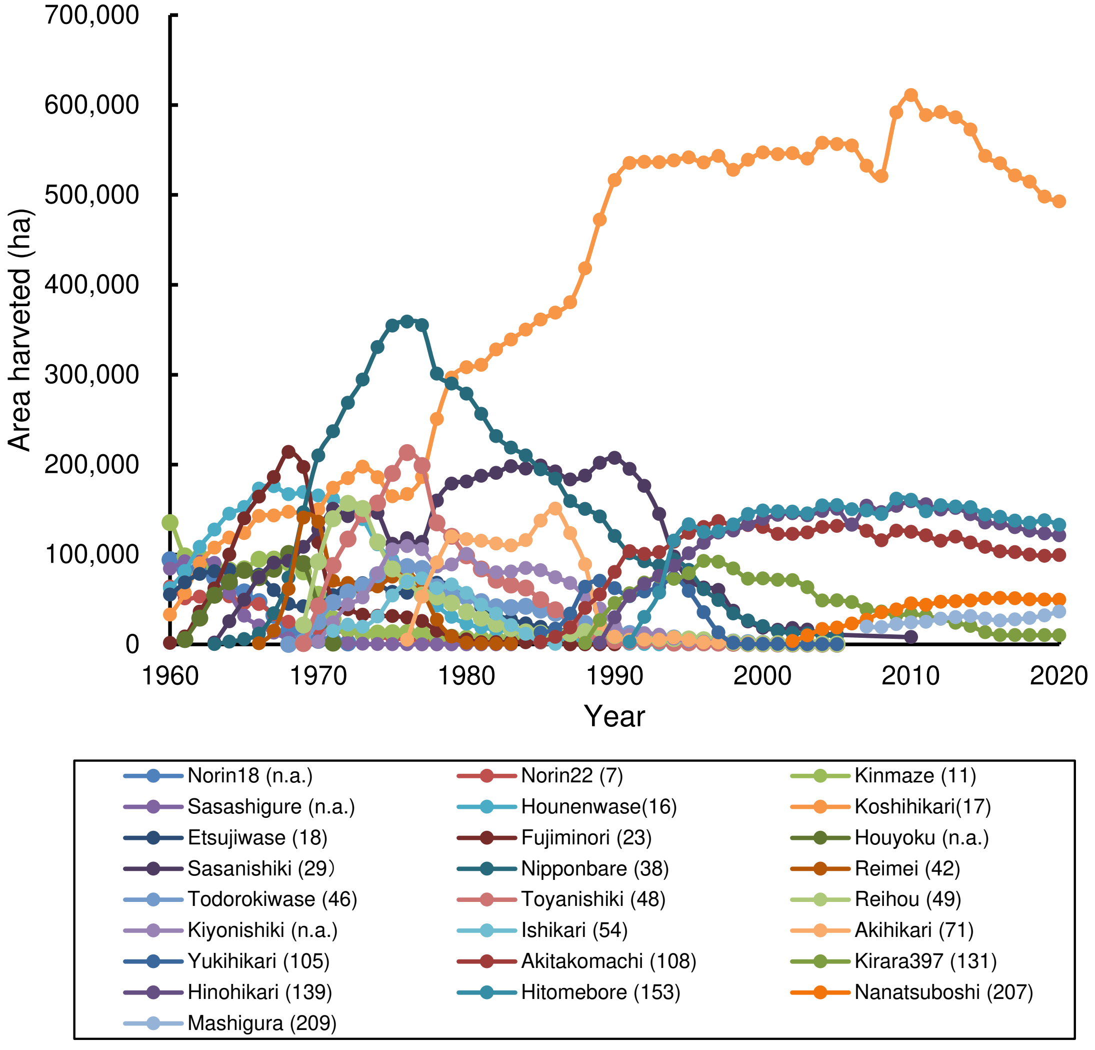

Supplementary Figure S11. Historical change in the area of cultivars grown in Japan from 1956 to 2020. The numbers in parenthesis after cultivar name refer to ID, which is the 237 core-cultivars and the 23 major cultivars (Supplementary Table S2 and S3), and "n.a." indicates cultivars not selected as the core-237 cultivars. Data from 1956 to 2005 is on Ministary of Agricutlure and Fisheries (http://www.library-archive.maff.go.jp/search?searchtype=category&start=1&rows=20&sort=title&category=153&categoryname=%E7%B1%B3%E7%A9%80%E3%81%AE%E5%93%81%E7%A8%AE%E5%88%A5%E4%BD%9C%E4%BB%98%E7%8A%B6%E6%B3%81), data from 2006 to 2020 is from Kome-Net (https://www.komenet.jp/data/jishuchousa/hinsyu/).

Fig.S12. Shimono

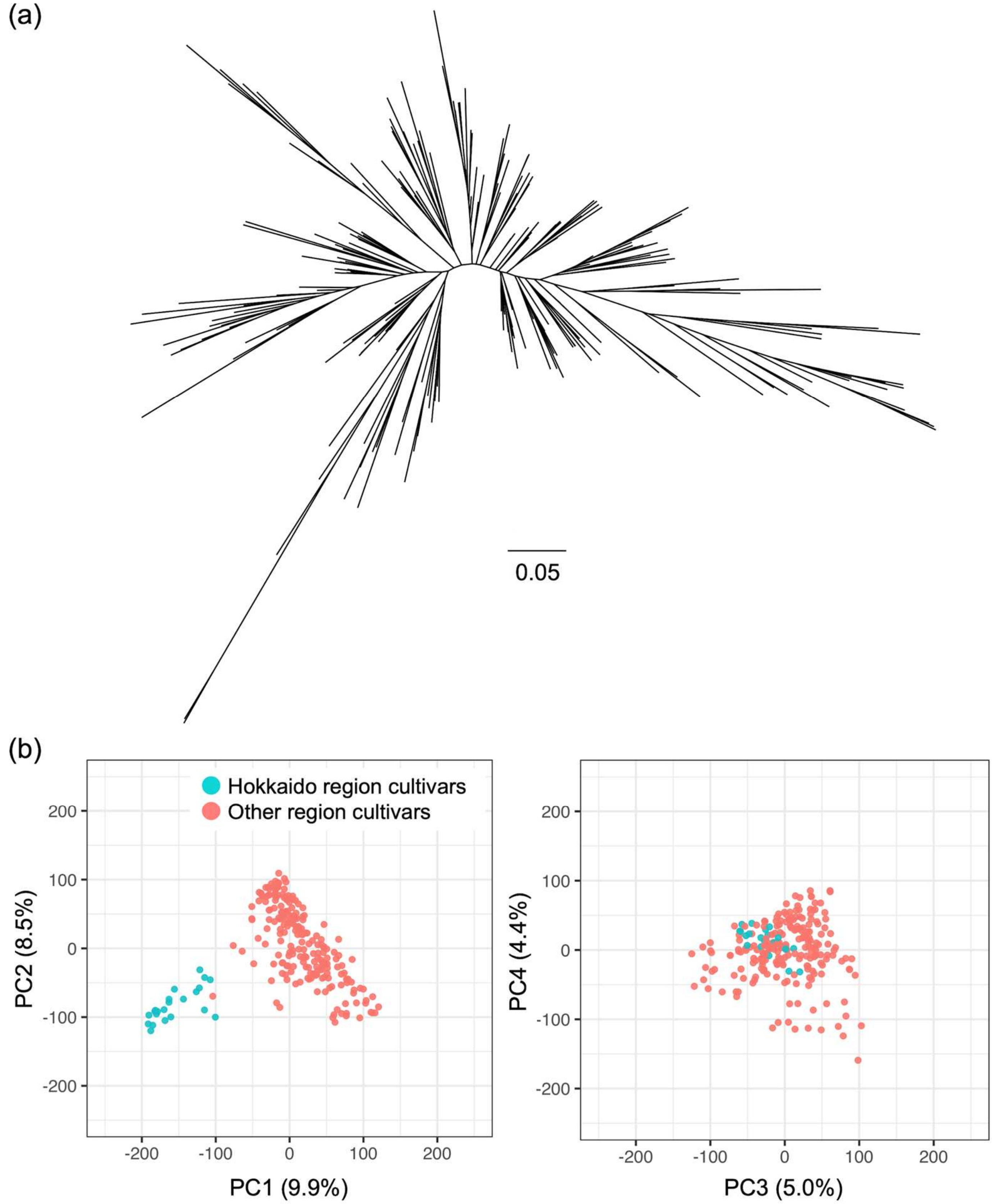

Supplementary Figure S12. Phylogenetic tree and Principal components analysis (PCA) based on whole-genome 91 800 SNPs during 237 core cultivars. (a) Midpoint-rooted Neighbor-Joining tree. The MEGA-CC 11 software (Kumar et al. 2021) was used to create a Neighbor-Joining tree. The program was executed using default parameters, and 1000 bootstrap replicates. The tree is drawn to scale, with branch lengths in the same units as those of the evolutionary distances used to infer the phylogenetic tree. FigTree v1.4.4 software (http://tree.bio.ed.ac.uk/software/figtree/) was used for visualization. (b) PCA plots of 237 core cultivars. Hokkaido region is the northern limit of rice cultivation in Japan (Supplementary Figure S2). PCA was performed using the R package pcaMethods (Stacklies et al. 2007) with the pca function with svd method and nPcs=4.

(a) Yield-plasticity (α)

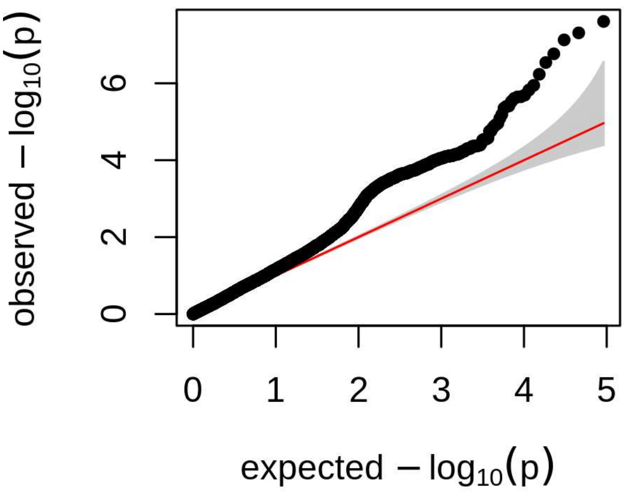

(b) Yield-ability (β) (t/ha)

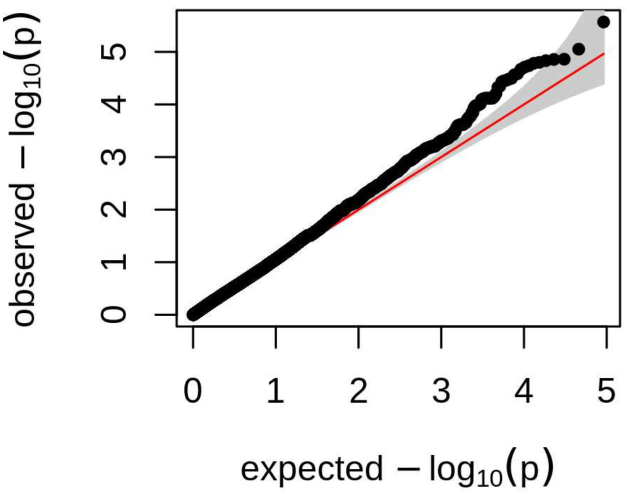

Supplementary Figure S13. Q-Q plots of GWAS for (a) yield-plasticity and (b) yield-ability. Q-Q plots were visualized by the R gaston package (Perdry and Dandine-Roulland 2018). The black dots represent the observed -log10(p) of each SNPs, and the red line is the expectation under the null hypothesis of no association. The grey band represent a 95% confidence interval.
