# Supplementary material for: Upcycling rice yield trial data using a weather-driven crop growth model
Source: Commun Biol. 2023 Jul 21;6:764. doi: 10.1038/s42003-023-05145-x (PMC10362053; doi:10.1038/s42003-023-05145-x)
Supplement: Supplementary file 3 — Description of Additional Supplementary Files [file 42003_2023_5145_MOESM3_ESM.pdf]

Description of Additional Supplementary Files

File name: Supplementary Data 1
Description: Source data of Figure 1ab.

File name: Supplementary Data 2
Description: Source data of Figure 2a.

File name: Supplementary Data 3
Description: Source data of Figure 2b.

File name: Supplementary Data 4
Description: Source data of Figure 3e.

File name: Supplementary Data 5
Description: Source data of Figure 4b.

File name: Supplementary Data 6
Description: Locations of the yield tests for the 237 core cultivars from 1980 to 2017, representing data from a total of 72 510 yield trials. ID numbers refer to the locations shown in Supplementary Figure S2..

File name: Supplementary Data 7
Description: Genopypic coefficients of the yield-plasticity (α, dimensionless) and yield-ability (β; t/ha) by YpCGM method, potential yield (Y_p) estimated by the crop growth model, and observed data of yield (Y_{obs}), and daily mean air temperature and solar radiation in trials of the 237 core cultivars, for a total of 72 510 datasets.

File name: Supplementary Data 8
Description: Root-mean-square error (*RMSE*) of the difference between the observed yield and the potential yield (Y_p), as well as the observed panicle number and panicle length, and the observed range of days to heading, panicle number, and panicle length.

File name: Supplementary Data 9
Description: The list of rice cultivars and DDBJ sequence read archive used in this study.
